# Measuring the coverage of nutrition interventions along the continuum of care: time to act at scale

**DOI:** 10.1136/bmjgh-2018-001290

**Published:** 2019-06-24

**Authors:** Stuart Gillespie, Purnima Menon, Rebecca Heidkamp, Ellen Piwoz, Rahul Rawat, Melinda Munos, Robert Black, Chika Hayashi, Kuntal Kumar Saha, Jennifer Requejo

**Affiliations:** 1 Poverty, Health and Nutrition Division, International Food Policy Research Institute, Washington, District of Columbia, USA; 2 International Health, Johns Hopkins University Bloomberg School of Public Health, Baltimore, Maryland, USA; 3 Global Development Program, Bill and Melinda Gates Foundation, Seattle, Washington, USA; 4 Monitoring and Statistics, Unicef USA, New York, New York, USA; 5 Nutrition for Health and Development, Organisation mondiale de la Sante, Geneva, Switzerland

**Keywords:** health systems, nutrition, prevention strategies, Child health, maternal health

## Abstract

The global community is committed to addressing malnutrition. And yet, coverage data for high-impact interventions along the continuum of care remain scarce due to several measurement and data collection challenges. In this analysis paper, we identify 24 nutrition interventions that should be tracked by all countries, and determine if their coverage is currently measured by major household nutrition and health surveys. We then present three case studies, using published literature and empirical data from large-scale initiatives, to illustrate the kind of data collection innovations that are feasible. We find that data are not routinely collected in a standardised way across countries for most of the core set of interventions. Case studies—of growth monitoring and screening for acute malnutrition, infant and young child feeding counselling, and nutrition monitoring in India—highlight both challenges and potential solutions. Advancing the nutrition intervention coverage measurement agenda is essential for sustained progress in driving down rates of malnutrition. It will require (1) global consensus on a core set of validated coverage indicators on proven, high-impact nutrition-specific interventions; (2) the inclusion of coverage measurement and indicator guidance in WHO intervention recommendations; (3) the incorporation of these indicators into data collection mechanisms and relevant intervention delivery platforms; and (4) an agenda for continuous measurement improvement.

**Summary box**

- High-quality actionable data are crucial for turning political commitment to scale up nutrition into visible results on the ground.
- We propose a set of high-impact nutrition-specific interventions along with indicators for tracking their coverage.
- A value chain approach to the generation, analysis, communication and use of data is key for progress.

## Introduction

Child and maternal undernutrition and poor diets are the top two risk factors for death and disability worldwide, accounting for 11.5% and 9.6% of disability-adjusted life years lost, respectively.^1 2^ The global community has committed to addressing malnutrition, as evidenced by several declarations and goals—including a set of six global nutrition targets, endorsed by the World Health Assembly (WHA) in 2012 and the second Sustainable Development Goal (SDG 2) which aims to end hunger and all forms of malnutrition by 2030.

To track progress towards these goals and targets, several nutrition-relevant monitoring and accountability frameworks and initiatives have emerged in recent years. For these initiatives to be effective in their accountability roles, a timely supply of valid, actionable data is essential. The multifaceted aetiology of malnutrition and the required multisectoral response means a range of data from multiple levels is required. Data that capture relevant information on distal factors such as policies and the regulatory environment, and on the health, food, education, and water and sanitation systems are key. Information on the coverage of nutrition programmes and variance in access to these programmes and in nutrition outcomes across different population groups is important.

In 2013, the Lancet Maternal and Child Nutrition Series recommended a package of nutrition interventions that, if scaled to 90% coverage, could reduce stunting by 20% and reduce infant and child mortality by 15%.^2^ Because intervention coverage changes more rapidly than nutritional status or mortality in response to policy and programmatic actions, routine monitoring of intervention coverage enables rapid assessment of progress and helps identify any need for mid-course corrections.^3^ Thus, monitoring the scale and quality of these interventions—now incorporated into many national policies and programs^4^—is essential for tracking country progress towards national and global goals.

Comparable, reliable coverage data for these interventions, however, are scarce due to several measurement and data collection challenges. To address these, the inaugural GNR recommends (1) using existing data better, (2) strengthening existing data collection processes, (3) improving data comparability across countries, (4) collecting new data where there are not enough for good accountability and (5) increasing the frequency of national nutrition survey data collection.^5^

Given the glaring gaps in data on nutrition interventions and their centrality to programme management and progress assessment, this paper focuses on intervention coverage—defined as the proportion of individuals in need of a service that actually receive the service. We focus on the coverage of interventions with proven impact on reducing undernutrition in women and children, mainly delivered through the health system. Our specific aims are to (1) assess data availability of a set of recommended nutrition interventions, (2) illustrate the challenges and opportunities for stronger measurement of these nutrition interventions through two case studies and a country example, and (3) propose a way forward to establishing a strong nutrition data ecosystem.

This paper is part of a Series that aims to address the challenges in measurement and monitoring women’s, children’s and adolescents’ health in the context of the sustainable development goals. The series includes improved ways to measure and monitor inequalities, drivers of women’s, children’s and adolescents’ health especially governance, early childhood development, reproductive maternal and child health in conflict settings, nutrition intervention coverage and effective coverage of interventions. These papers were developed as part of an initiative of the multi-institutional Countdown to 2030 for women’s, children’s and adolescents’ health, presented at a Countdown measurement conference 31 January to 1 February 2018 in South Africa, and reviewed by members of the Countdown working groups.

## Data availability for a core set of nutrition-specific interventions

We first identified a core set of proven nutrition interventions that should be tracked across all countries and determined if coverage of each of them is currently measured in USAID-supported Demographic and Health Surveys (DHS) and Unicef-supported Multiple Indicator Cluster Surveys (MICS), the two major household survey programmes carried out in low-income and middle-income countries. To do this, we combined the recommended list in the Lancet Maternal and Child Nutrition Series^2^ with current WHO global guidance for nutrition-specific interventions that can be feasibly delivered in low-income and middle-income countries (http://www.who.int/publications/guidelines/nutrition/en/ and/or from WHO’s Electronic Library of Evidence-based Nutrition Actions, http://www.who.int/elena/en/, both reviewed on 30 January 2018). These interventions are primarily delivered by the health sector through facility or community service delivery channels. Interventions delivered mainly through agri-food (eg, food fortification), school (eg, mid-day meals, nutrition education) or other non-health systems were excluded.

To assess data availability of these interventions, we reviewed current questionnaires for the DHS (https://dhsprogram.com/publications/publication-dhsq7-dhs-questionnaires-and-manuals.cfm) Phase 7 2013–2018 and the MICS (MICS 6 revised in 2017; http://mics.unicef.org/tools). Together, these surveys cover over 100 countries with multiple waves of data collection every 3–5 years, and make the dominant contribution to global databases on health and nutrition. We reviewed all questionnaires (household, woman and child) and documentation for both surveys. For each intervention for which data were available, we considered measurement issues related to respondents, recall periods, questions and response codes.

Our working list of evidence-based nutrition interventions delivered through the health system, by phase along the continuum of care, is shown in table 1. Against each intervention, we note whether coverage is ascertained in the core modules of the two main household surveys (DHS and MICS), the degree to which collected information can be used to form useful coverage indicators, along with other key measurement considerations.

**Table 1:** Proposed indicators for a core set of effective nutrition interventions delivered through the health system, by phase along the continuum of care

Intervention	Data availability (core DHS 7 and/or MICS 6)	Potential indicator definition
Preconception*		
Iron supplementation (W)	No	Percentage of non-pregnant women ages 15–49 who received ANY iron containing supplements in the last X months
Folic acid supplementation (L, W)	No	Percentage of non-pregnant women ages 15–49 who received ANY folic acid containing supplements in the last X months
Pregnancy		
Any nutrition counselling during pregnancy (W)	No	Percentage of women who received ANY information by ANY provider about diet or physical activity during pregnancy
Nutrition counselling during pregnancy (specific content) (W)	No	Percentage of women who received information on the following topics during pregnancy: physical activity, diet (quality and quantity), micronutrients, breast feeding, other
Balanced energy protein supplementation (L, W)	No	Percentage of women meeting criteria for need that received any food or macronutrient supplements during pregnancy
Iron–folic acid supplementation (IFA) (L, W)	Yes, DHS 7. Questions do not distinguish composition of supplement beyond ‘iron containing’. Long period for maternal recall is a concern for validity	Percentage of women who received any IFA during pregnancy
Multiple micronutrient (MMN) supplementation (L)	No. They may qualify as ‘iron-containing’ supplement in DHS 7 but there is no way to distinguish it was a MMN supplement.	Percentage of women who received any MMN supplements during pregnancy
Calcium supplementation for pregnant women with low calcium intakes (L, W)	No	Percentage of women with low calcium intakes who received any calcium supplements during pregnancy
Vitamin A supplementation (low dose for populations with high prevalence of deficiency) (W)	No	Percentage of women in populations at risk of deficiency who received any low-dose vitamin A supplements during pregnancy
Deworming for populations where pregnant women have a 20% or higher prevalence of infection with hookworm or *T. trichiura* infection AND a 40% or higher prevalence of anaemia (W)	Yes, DHS 7. Concern that women may not be able to recall differences between drugs provided for deworming, IPTp or iron and IFA supplements	Percentage of women in targeted areas who received any albendazole during pregnancy
Around delivery/postnatal		
Delayed cord clamping (W)	No	Percentage of women whose cord was clamped at 2 min after birth
Support for early initiation of breast feeding (L, W)	Yes, DHS 7 and MICS 6. Survey questions are included in the postnatal care module so time period is limited to any time in the first 2 days after childbirth	Percentage of women who were assisted by a provider to put the infant to the breast in the first hour after childbirth
Iron supplementation (lactating women) (W)	No	Percentage of lactating women who received any iron-containing supplement within 6 months after childbirth
Childhood prevention and promotion		
Support for exclusive and continued breast feeding (L, W)	Yes, DHS 7 and MICS 6. Survey questions are included in the postnatal care module so time period is limited to any time in the first 2 days after childbirth. Support may be needed later on during infancy	Percentage of women with a child 0–6 months of age who received advice/information from a healthcare provider or community worker within 1 month after childbirth; and/or percentage of women with a child 0–6 months of age who were observed breast feeding by a health provider or community. *Additional time periods after childbirth may be added depending on country/context-specific services*
Counselling for exclusive and continued breast feeding (L, W)	Yes, DHS 7 and MICS 6. Survey questions are included in the postnatal care module so time period is limited to any time in the first 2 days after childbirth. Information/counselling may be needed later on during infancy	Percentage of women with a child 0–6 months of age who received information/counselling about exclusive breast feeding from a health provider or community worker in the last 6 months
Counselling for complementary feeding (L, W)	No	Percentage of women with a child 6–24 months of age who received information from a provider about key components of complementary feeding within the previous X months, including timing of introduction of semisolid and solid foods, diet diversity and other local messages?
Food supplementation for complementary feeding in food-insecure populations (L, W)	Yes, DHS 7. Questions ask only about ready-to-use supplemental foods with 7-day recall period. Questions are not currently located with the IYCF practice questions	Percentage of children 6–23 months of age from food-insecure populations who received any food supplements in the last X months
Iron supplementation in a population where children of 6–59 months have 20% or higher prevalence of anaemia (W)	No	Percentage of children (6–59 months) in a selected population who received any iron supplements in the X days before the survey Percentage of children (6–59 months) in a selected population who received at least X doses of iron supplements in the X days before the survey
Vitamin A supplementation (high dose)† (L, W)	Yes, DHS 7. Questions ask about a single dose in the previous 6 months	Percentage of children (6–59 months) who received a high-dose vitamin A supplement in the 6 months preceding the survey
Multiple micronutrient powders (MNP for anaemia) in a population where children of this age have 20% or higher prevalence of anaemia (L, W)	Yes, DHS 7. Questions use a 7-day recall period, which is limited to current consumption	Percentage of children (6–59 months) in a selected population who received *any* MNP in the last X days prior to the survey Percentage of children (6–59 months) in a selected population who received at least N doses of MNP in the last X days prior to the survey
Preventive zinc supplementation (L)	No	Percentage of children (6–59 months) who received any preventive zinc supplementation in the X days before the survey
Childhood treatment		
Management of severe acute malnutrition (SAM) (L, W)	Yes, DHS 7. Questions ask only about ready-to-use therapeutic foods with 7-day recall period. Challenge is on identifying the appropriate population of children (the denominator)	Percentage of children 6–59 months of age who are identified as having SAM that received treatment (a special food supplement)
Management of moderate acute malnutrition (MAM) (L, W)	Yes, DHS 7. Questions ask only about ready-to-use supplemental foods with 7-day recall period. Challenge is on identifying the appropriate population of children (the denominator)	Percentage of children 6–59 months of age who are identified as having MAM that received treatment (a special food supplement)
Zinc supplementation with oral rehydration salts (ORS) for children with diarrhoea (L, W)	Yes, DHS 7 and MICS 6. Both survey programmes use an aided recall of receipt of zinc	Percentage of children who received zinc and ORS for an episode of diarrhoea in the 2 weeks before the survey

*‘The preconception phase’ includes interventions delivered during the time period prior to a first pregnancy and interpregnancy intervals as well as interventions provided to women of reproductive age and adolescent girls (ages 10–14) who do not eventually or ever become pregnant.

†Unicef includes in its databases the following indicator which is based upon a combination of survey and administrative data: percentage of children ages 6–59 months who received two doses of vitamin A during the calendar year.

DHS, Demographic and Health Survey; IPTp, Intermittent preventive treatment in pregnancy; IYCF, infant and young child feeding; L, Included in the Lancet Maternal and Child Nutrition Series, 2013; MICS, Multiple Indicator Cluster Survey; W, recommended by WHO.

Our analysis shows that data are not routinely collected in a standardised way across countries for most of these interventions. The DHS and/or MICS include relevant questions for less than half of the recommended interventions, and even these questions may not be adequate for constructing actionable coverage indicators (as highlighted in column 2 of table 1). Although DHS and MICS are regularly reviewed, our review suggests that current questionnaires have not been systematically updated to reflect current nutrition recommendations. These shortcomings are due in part to the absence of consolidated global guidance on SMART (‘specific, measurable, achievable, relevant and time-bound’) indicators for recommended nutrition interventions.

## Measurement case studies

Using published literature and empirical data from large-scale initiatives, we present three case studies of measurement challenges and solutions. The first two focus on growth assessment and counselling—two of the three main types of nutrition activities currently being implemented at country level (as per WHO 2018) for which recent efforts have been introduced to improve their measurement (the third being micronutrient supplementation which has a stronger literature base).^2^ The third case study highlights how one country—India—has modified its data collection tools to better collect information on these two key intervention areas.

### Case 1: growth monitoring and screening for acute malnutrition

#### Why is this important, and what has been done?

Monthly assessment of infant and young children’s weight and height (or length), plotted on a standardised chart, also known as growth monitoring (GM), is a cornerstone activity of paediatric clinical care and some community-based programmes.^4^ GM is an entry point for delivery of nutrition counselling and other preventive interventions.

Screening for acute malnutrition involves taking an anthropometric measurement—typically mid-upper arm circumference (MUAC) or weight-for-height Z-score—comparing it with a threshold value and assessing oedema status to determine whether to refer a child into a treatment programme.^6^ GM and screening for acute malnutrition may happen at the same point of contact in settings where acute malnutrition interventions are integrated into routine services.^7 8^ Screening, however, is often done separately in campaign-style outreach, especially in settings of acute food insecurity or humanitarian crisis.

#### Challenges and responses

Although GM and screening for acute malnutrition are commonly implemented nutrition activities, their population-based coverage is not reported across low-income and middle-income countries. Questions about these activities are not included in core DHS or MICS questionnaires. In administrative systems, GM and screening activities may be tracked through individual register books but typically only referrals into acute malnutrition treatment programmes and treatment outcomes are compiled and reported to higher levels. This is a missed opportunity as monitoring coverage of ‘entry point’ activities can help explain why other interventions are not reaching target groups. The number of children meeting the criteria for treatment, regardless of whether they actually received treatment, is needed for determining the target population (the population in need of the service or the denominator) which is essential for accurately assessing coverage levels of acute malnutrition treatment.

The absence of systematic research on recall by the caregiver (usually the mother) of GM or nutritional assessment activities represents another challenge. In 2017, the Performance Monitoring and Accountability 2020 (PMA2020) survey programme included questions on caregiver recall of assessment of weight, length and MUAC in the previous month among children 0–59 months in nationally representative household surveys in Kenya and Burkina Faso. Eligible households (4628 in Kenya, 2283 in Burkina Faso) were those with at least one child under 2 years and a woman of reproductive age. In Kenya, 54.9% reported *at least one* of the three measurements in the previous 30 days compared with 34.8% in Burkina Faso. However, children in Burkina Faso were more likely to have had all three indicators assessed in the previous 30 days (17.9% compared with Kenya’s 8.8%, table 2). In both contexts, the proportion of children measured by any method decreases or stagnates as they age—a pattern that is consistent with children having fewer contacts with the health system after they finish the vaccination schedule and move beyond nutrition interventions targeted to children under 2 years. The PMA2020 experience suggests it is feasible to measure coverage of specific nutritional assessment activities through caregiver report, although a validation study of maternal recall compared with a confirmed record of the activity is still needed.

**Table 2:** Methods of growth assessment in last 30 days among children 0–59 months in Kenya and Burkina Faso, national estimate, PMA2020 (2017)

	Burkina Faso n=3729 (%)	Kenya n=6434 (%)
Weight only	3.4	24.9
Height only	0.2	0.6
MUAC only	1.3	0.2
Weight and height	10.4	19.7
Weight and MUAC	0.9	0.7
Height and MUAC	0.2	0.05
Weight, height and MUAC	17.9	8.8
Total (any of the 3)	34.8	54.9

MUAC, mid-upper arm circumference; PMA2020, Performance Monitoring and Accountability 2020.

The ideal indicator for programme monitoring and evaluation will depend on the intended use of the data and country-specific guidelines that define which measurements should be taken and at what interval. Generally, ‘the proportion of children with at least one measurement in the previous 30 days’ will give a high-level snapshot of the nutritional assessment activities for global monitoring purposes. However, countries may prefer a more specific measure (eg, the proportion with MUAC assessment in the previous 30 days) that corresponds to their policies.

### Case 2: infant and young child feeding (IYCF) counselling

#### Why is this important, and what has been done?

Most countries (surveyed in WHO 2018) noted that they implement programmes to support breast feeding and complementary feeding. Yet, little is known about their reach and scale given the absence of consensus on a set of indicators to capture programme coverage. Instead, many global monitoring efforts report on IYCF practices (eg, exclusive breast feeding) instead of IYCF intervention coverage.

Most prior experience measuring IYCF programme coverage came from intervention studies where coverage was defined by measuring exposure reported by mothers to specific programme messages and/or job aids and visual materials.^9–11^ Insights from such efforts were adapted for use in the impact and process evaluations of large-scale programmes delivered by *Alive and Thrive* (A&T), an initiative to demonstrate impact of programmes to improve IYCF practices in several countries.

Because IYCF practices vary by context (including geographic, social, economic and individual), programmes need to be appropriately tailored to reach mothers and communities. Across programmes, there may be variability in *platforms* (including types of frontline workers and delivery channels), *content* (specific behaviours that are promoted) and *frequency of contact*. Programme evaluations that aim to link exposure to outcomes will characterise coverage by measuring exposure via all of these dimensions, making cross-country comparisons and global assessments of progress challenging.

**Figure 1:**
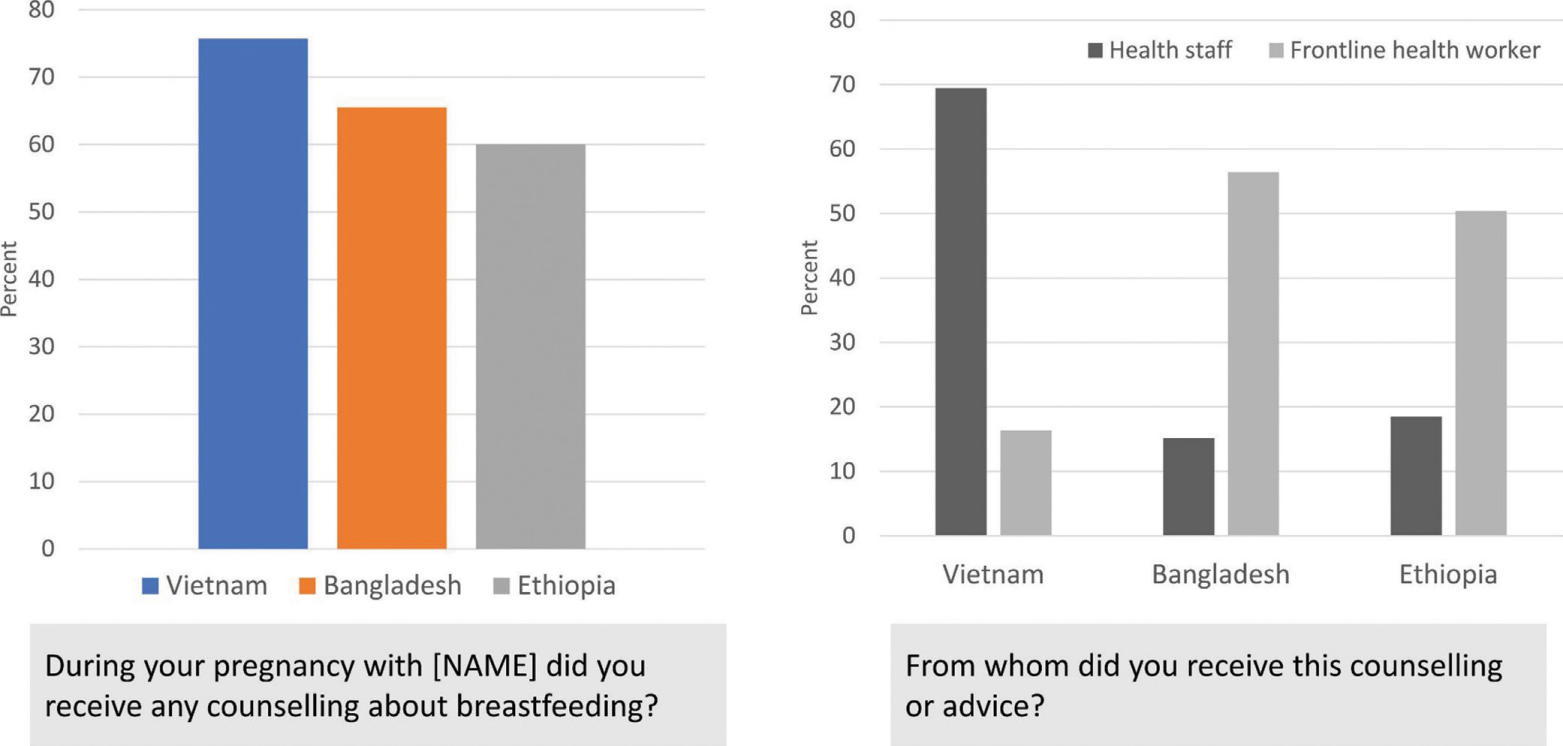
Illustration of the use of core questions about counseling on breast feeding during pregnancy in Bangladesh, Vietnam and Ethiopia.^16 17^

In the A&T programmes, for example, key messages were delivered via health workers (counselling during at home visits or in health facilities), mass media (TV or radio) and through social mobilisation activities (to reach fathers, other community members). Programme evaluations included questions that ascertained service contact, exposure to messages and exposure frequency. They often relied on programme-specific elements to encourage recall (eg, frontline worker name or shirt colour; specific visual aids). Figure 1 shows findings from three countries where evaluations were carried out. The results were consistent with what was expected from programme designs, suggesting that information can be recalled correctly, although formal validation studies need to be undertaken.

#### Challenges and responses

The A&T experience suggests that it is feasible to design and develop measures of exposure that align with intervention contexts and content of messages. What is needed is a core set of questions to be included in larger nationally representative surveys that capture exposure to counselling and support interventions for infant feeding. The DHS began this process by including questions on postnatal support for breast feeding in the DHS-6 questionnaire, but this needs further expansion to cover exposure during the early initiation, exclusive breast feeding and complementary feeding periods. Individual countries can adapt the core DHS questionnaire to measure exposure to their nutrition programmes (see India example below). Given that counselling is rarely delivered just once, measures that account for the cumulative nature of intervention delivery/exposure are needed—as are measures of the quality of counselling.

### Case 3: capturing coverage of counselling and food supplementation interventions in India

Adaptations to the core DHS instrument in India (the National Family Health Survey or NFHS) provide one example of how countries can enable more country-relevant tracking of nutrition coverage. India’s policy framework for nutrition includes most evidence-based nutrition interventions.^12^ In the context of major policy changes related to health and nutrition between 2006 and 2016, data from India’s NFHS demonstrate a rising trend in the coverage of nutrition interventions across the continuum of care.^13^

**Figure 2:**
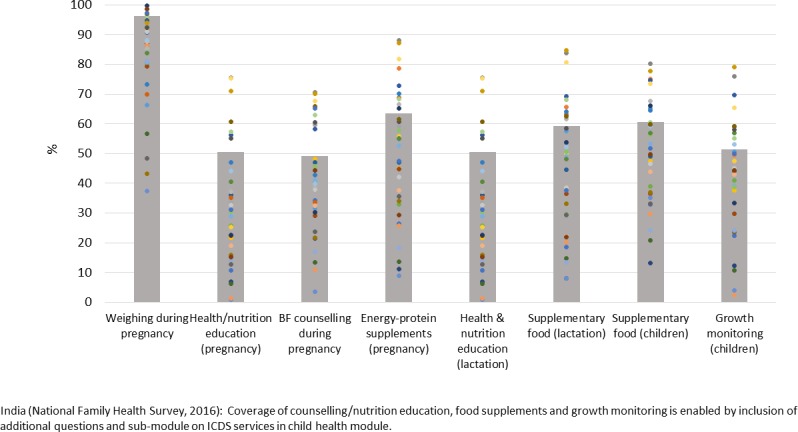
Average national (bars) and state-level (dots) coverage of key nutrition actions (counselling, growth monitoring and food supplements) typically not included in the core Demographic and Health Survey and Multiple Indicator Cluster Survey questionnaires. BF, breast feeding; ICDS, Integrated Child Development Services.

The NFHS includes questions on health and nutrition counselling, food supplementation and growth monitoring within a special submodule on the Integrated Child Development Services (ICDS; India’s flagship nutrition programme). With a focus on all children born to the respondent woman in the last 5 years, questions relate to the types of ICDS services received in the last 12 months, well beyond the coverage analyses shown in figure 2. Such questions could be even more valuable if expanded to include follow-up questions on the content of counselling or on actions taken after growth monitoring (advice and/or referrals). There is a need, however, to guard against possible confusion due to duplication (eg, where questions about nutrition components of antenatal care such as weight monitoring and breastfeeding counselling are also included in the antenatal care (ANC) module). Finally, since food supplementation is a major cost component of India’s nutrition programmes,^14^ inclusion of questions on acceptability of ICDS food supplements could help strengthen such programmes.

India’s new National Nutrition Mission (launched on March 2018) is already using this expanded set of coverage indicators to assess baseline levels and monitor district performance (including planned surveys in focus districts) as the programme unfolds (http://niti.gov.in/content/nutrition-charts). The Ministry of Health and Family Welfare’s MIS provides information on some nutrition actions (eg, nutritional counselling in ANC, micronutrients) while the Ministry of Women and Child Development’s ICDS MIS provides data on others (eg, food supplements, growth monitoring). Management of the Mission’s monitoring and data systems by two separate ministries, however, will raise challenges for coverage tracking—for example, the lack of alignment of beneficiary catchment areas and different approaches to capturing identification information.

## Conclusions

Data have many purposes—to define and characterise different types of nutrition problems, to highlight magnitude, distribution and variability, over time and space; to understand what is driving the problem; to design, deliver and monitor appropriately targeted interventions and determine their effectiveness; to track national and global levels and trends; and to hold responsible actors accountable for progress (or lack thereof) towards goals they have signed up to. Without relevant and timely data, we are at best myopic, and at worst, flying blind. We need better data for use, and we need better use of data.

Since the call for a ‘nutrition data revolution’ in the first GNR in 2014, some progress has been made in driving a more systemic ‘data value chain’ approach that sequentially links priority setting with the collection, curation, analysis, interpretation and use of appropriate data to inform decisions on action.^15^

In this paper, we have examined one major component of the nutrition data challenge—the lack of consensus on indicators of coverage of a core package of interventions to be tracked across available data platforms in countries with high burdens of maternal and child undernutrition. We have highlighted both challenges and potential solutions, with specific case studies.

We started by developing a list of key nutrition-specific interventions that can be delivered through the health system, and for which global guidelines exist (table 1). We then proposed a list of indicators that capture information on coverage of these interventions, highlighting specific considerations in their measurement. We recognise that several of these interventions are not being implemented in countries and that some are lower priority than others.

Advancing the nutrition intervention coverage measurement agenda is foundational for addressing the wider nutrition data challenge. It will require:

1. A process for generating global consensus on a minimum core set of validated coverage indicators (as proposed in table 1) on proven, high-impact nutrition-specific interventions.
2. The inclusion of coverage measurement and indicator guidance in WHO intervention recommendation and guidance documents.
3. The incorporation of these indicators into data collection mechanisms (via revisions of survey questionnaires and routine nutrition and health information systems) as well as relevant intervention delivery platforms. Such a process would require the questions highlighted in box 1 to be addressed.
4. An agenda for continuous measurement improvement, including indicator validation and means for adjusting coverage estimates to take into account delivery quality and effectiveness (effective coverage).

Box 1 **Nutrition intervention measurement considerations**

Here, we highlight some important considerations to ensure effective measurement of nutrition intervention coverage. Indicator-specific measurement considerations are discussed in the online supplementary table 1.

- *Which effective interventions are useful to monitor at global and country level (and therefore require population-based measures)?*
- *Is the intervention defined in a relatively consistent way across countries, such that a global indicator and set of survey questions can be defined?* The survey questions for many indicators require some degree of country-level adaptation—for example, local brands of processed foods, fruits and vegetables, or local cadres of health workers need to be specified. If the intervention itself changes significantly from country to country, however, it may be challenging to define an indicator that can be measured in a standard way across countries.
- *How large is the population in need of the intervention? What is an appropriate reference period for the intervention?* A typical DHS or MICS samples approximately 10 000–15 000 households in most countries, although some, like India and Nigeria, have much larger samples. The effective sample size is reduced because of the use of cluster sampling, which inflates the variance of survey-based indicators. Interventions targeting relatively rare conditions may not be measurable in population-based surveys because it is not possible to identify a sufficient number of individuals in need of the intervention. In some cases, the reference period can be lengthened to identify a greater number of individuals in need—for example, with interventions for pregnancies and deliveries, where questions are asked about completed pregnancies over several years preceding the survey. The sample size benefits of increasing the recall period, however, need to be balanced against the risk of eroding recall over time. If a biomarker is used to establish need, the reference period may need to be very short—for example, in the case of severe acute malnutrition where measurement of weight and length can identify currently malnourished children, but not previous cases of malnutrition.
- *Can surveys accurately capture whether respondents needed the intervention?* Intervention coverage should be measured using denominators that reflect the population in need of the intervention. This can be relatively straightforward for preventive interventions targeted by age, sex or pregnancy status, but is more complex for curative interventions that require a diagnosis. For example, to measure coverage of management of acute malnutrition, a survey would need to identify all children with acute malnutrition during a predefined period. Where appropriate diagnostic biomarkers exist, they may be included in surveys and used to determine the target population.
- *Can survey respondents report accurately on whether they received the intervention?* A number of factors may affect reporting of interventions. Respondents may not recall (or may not have been told) the name of the treatment given or the reason the treatment was given. Interventions that require questions about adherence (eg, iron folic acid supplementation in pregnancy), timing (eg, early initiation of breast feeding) or messages (eg, counselling interventions) have more opportunities for error in reporting. Interventions delivered during sensitive or high-stress times, such as the intrapartum and immediate postnatal periods, may also be difficult to report on accurately.^18 19^
- *Can the intervention be measured in a health facility assessment?* Health facility assessments (HFAs) allow for direct measurement of the environment in which services are delivered including readiness to provide services and in some cases the quality of case management, and thus provide information that cannot be obtained from household surveys. HFAs typically assess services only within facilities and therefore cannot measure community-based and other non-facility interventions. In addition, they may not have sufficient sample size to capture interventions for rare conditions, and assessing the quality of case management can be challenging. Being facility-based, HFAs do not provide population-based measures of intervention coverage, although in some cases it may be possible to estimate a denominator. However, household surveys can be combined with concurrent or recent HFAs to obtain coverage estimates for interventions delivered through health facilities that are not measurable in household surveys.
- *Can the intervention be measured in the NHIS? Is it currently measured?* For interventions to be reported through a country’s National Health Information System (NHIS), they must be recorded in standardised consultation registers or medical records and an item included in the quarterly NHIS report. In many countries, the NHIS reports only on a subset of interventions delivered through public health facilities, and the process of adding a variable to the NHIS may be lengthy. In addition, NHIS do not directly measure the population in need of the intervention, though this can sometimes be estimated from census or other population-based data, particularly for preventive interventions. Nonetheless, there is increasing interest in using NHIS and other administrative data to estimate intervention coverage, as they provide more timely estimates than household surveys or HFAs, at much lower cost. This is already being done for vitamin A supplementation of young children, where programme monitoring and NHIS data are being used to estimate intervention coverage.^20^

10.1136/bmjgh-2018-001290.supp1 Supplementary data

More broadly, accelerating progress towards the SDGs also calls for:

1. The adoption of a value chain approach to data, which views the entire ecosystem, encompassing an integrated and interoperable set of hardware, software, data, people and procedures that produces relevant data, that translates data into useful information and (via communication) into improved knowledge for action.
2. In-country mechanisms for priority-setting (taking into account local relevance and the cost of adding indicators into surveys), and for coordination of the collection and use of high-quality, timely data.
3. Operational guidance for data prioritisation, harmonisation of indicators and consistent incorporation of nutrition into routine management information systems.
4. Development of national data plans that are well costed, resourced and implemented over the long term (including financing for updating and maintaining national and global databases).
5. Strengthened national capacity for appropriate analysis of data (including disaggregation by equity stratifiers).
6. Implementation research, innovation and learning across the data value chain.
7. A strengthened enabling environment to support a culture for data and evidence use for planning and action. Data need to be persuasively communicated to policy-makers and programme managers in a way that facilitates action. Enabling policy and institutional environments also need to be built on strengthened commitment, governance, capacity and leadership at all levels.
8. The capture and dissemination of tacit and experiential knowledge (eg, ‘stories of the data revolution’) to both inform and inspire change.

This paper calls for accelerating coverage measurement of a prioritised set of nutrition interventions—delivered primarily through health systems—as a means for tracking programme progress, performance and accountability. Further efforts are needed to improve measures of effective coverage of nutrition interventions which would include considerations of the quality of the services delivered and their impact on nutritional status. Further work is also needed on measurement of nutrition in routine administrative data systems, and effective interventions delivered via other sectors and programmes.
