# Supplementary material for: Measuring the coverage of nutrition interventions along the continuum of care: time to act at scale
Source: BMJ Glob Health. 2019 Jun 24;4(Suppl 4):e001290. doi: 10.1136/bmjgh-2018-001290 (PMC6590959; doi:10.1136/bmjgh-2018-001290)
Supplement: Supplementary data [file bmjgh-2018-001290supp001.pdf]

ONLINE SUPPLEMENTARY TABLE (11 January 2019)

**Annex 1: Measurement considerations for proposed indicators of a core set of effective nutrition interventions delivered through the health system, by phase along the continuum of care**			
**Intervention**	**Data availability (core DHS7 and/or MICS6)**	**Potential indicator definition**	**Measurement considerations for inclusion in household surveys**
**PRECONCEPTION****			
Iron supplementation (W)	No	Percentage of non-pregnant women ages 15-49 who received ANY iron containing supplements in the last X months	• Denominator issues:<br>○ Decide whether data on adolescent girls ages 10-14 should/can be collected<br>○ Decide upon exclusion of lactating women<br>• Define supplement type (including if IFA, tablet or syrup)<br>• Decide whether to include questions on quantity of pills/syrup received<br>• Consider additional indicators to capture consumption of iron received<br>• Determine time period (i.e., in the last 6 months)
Folic acid supplementation (L,W)	No	Percentage of non-pregnant women ages 15-49 who received ANY folic acid containing supplements in the last X months	• Denominator issues:<br>○ Decide whether data on adolescent girls ages 10-14 should/can be collected<br>○ Decide upon exclusion of lactating women<br>• Define supplement type (including if IFA, and if consumption of fortified food should be considered)<br>• Decide whether to include questions on quantity of pills/syrup received<br>• Consider additional indicators to capture consumption of folic acid received<br>• Determine time period (i.e., in the last 6 months?)
**PREGNANCY**			
Any nutrition counseling during pregnancy (W)	No	Percentage of women who received ANY information by ANY provider about diet or physical activity during pregnancy	• Define recall period (e.g., most recent pregnancy within last X years)<br>• Define sources of information<br>• Determine how much detail to capture on the type of providers and/or platforms through which services were delivered (e.g., ANC, CHW)
Nutrition counseling during pregnancy (specific content) (W)	No	Percentage of women who received information on the following topics during pregnancy:<br>• Physical activity<br>• Diet (quality and quantity)<br>• Micronutrients<br>• Breastfeeding<br>• Other	• Define recall period (e.g., most recent pregnancy within last X years)<br>• Define sources of information<br>• Define specific messages/topics based on WHO or national guidelines<br>• Determine how much detail to capture on the type of providers and platforms through which services were delivered (e.g., ANC, CHW)
Balanced energy protein supplementation (L, W)	No	Percentage of women meeting criteria for need that received any food or macronutrient supplements during pregnancy	• Denominator issues<br>○ Criteria for need requires identifying pregnant women based on a poverty cut-off, experiencing food insecurity, and low BMI (<18.5 kg/m^2)<br>○ Identifying pregnant women with low BMI is not possible in retrospect (i.e., if women are asked about supplementation during or after a pregnancy)

			• Define recall period (e.g., most recent pregnancy within last X years)<br>• Determine if separate questions are needed on the type of food/supplements received<br>• Determine if separate questions are needed on the duration of the supplementation (e.g., for X months)<br>• Consider additional questions to capture consumption of food/supplements received
Iron-folic acid supplementation (IFA) (L,W)	Yes, DHS7.<br>Questions do not distinguish composition of supplement beyond "iron containing". Long period for maternal recall is a concern for validity.	Percentage of women who received any IFA during pregnancy	• Determine if IFA or "iron-containing" supplement<br>• Decide whether to include questions on quantity of pills/syrup received<br>• Consider additional indicators to capture consumption of pills/tablets received (e.g., for X days or more)<br>• Define recall period (e.g., most recent pregnancy within last X years)
Multiple micronutrient supplementation (L)	No<br>They may qualify as "iron-containing" supplement in DHS7 but there is no way to distinguish it was a MMN supplement.	Percentage of women who received any multiple micronutrient supplements (MMS) during pregnancy	• Confirm minimum definition of MMS (e.g., 3 or more plus iron) and whether multiple micronutrient powders are included<br>• Decide whether to include questions on quantity of pills received<br>• Consider additional indicators to capture consumption of pills received (e.g., for X days or more)<br>• Define recall period (e.g., most recent pregnancy within last X years)
Calcium supplementation for pregnant women with low calcium intakes (L, W)	No	Percentage of women with low calcium intakes who received any calcium supplements during pregnancy	• Denominator issue:<br>○ Challenge of identifying which populations have low calcium intakes<br>• Decide whether to include questions on quantity of pills received (Calcium supplementation involves consumption of multiple pills per day)<br>• Consider additional indicators to capture consumption of pills received (e.g., for X days or more)<br>• Define recall period (e.g., most recent pregnancy within last X years
Vitamin A supplementation (low-dose for populations with high prevalence of deficiency) (W)	No	Percentage of women in populations at risk of deficiency who received any low-dose vitamin A supplements during pregnancy	• Denominator issues:<br>○ Requires specific biochemical, clinical or dietary indicators of vitamin A status to classify population as areas where VA deficiency is a public health problem<br>• Daily (up to 10,000 IU) or weekly (up to 25,000 IU) dosing – unclear when to start<br>• Consider additional indicators to capture consumption of pills received (e.g., for X days/weeks or more)<br>• Define recall period (e.g., most recent pregnancy within last X years.
Deworming for populations where pregnant women have a 20% or higher prevalence of infection with hookworm or T trichiura infection AND a 40% or higher prevalence of anemia (W)	Yes, DHS 7.<br>Concern that women may not be able to recall differences between drugs provided for deworming, IPTp, or iron and IFA supplements.	Percentage of women in targeted areas who received any albendazole during pregnancy	• Denominator issues:<br>○ Defining populations with a 20% or higher prevalence of hookworm or T trichiura infection and a 40% or higher prevalence of anemia<br>• Challenge with distinguishing deworming drugs from other pills received (e.g., IPTp)
**AROUND DELIVERY / POSTNATAL**			
Delayed cord clamping (W)	No	Percentage of women whose cord was clamped at 2 minutes after birth	• Difficult to capture through HH surveys and at the individual level (because difficult for women to recall the 2-minute interval immediately after childbirth).<br>• Assessments can be made among service providers (as part of supervision) and as part of national policy reviews
Support for early initiation of breastfeeding (L, W)	Yes, DHS-7 and MICS 6.<br>Survey questions are included in the post-natal care module so time period is limited to any	Percentage of women who were assisted by a provider to put the infant to the breast in the first hour after childbirth.	• Can be problematic for women to determine the 1-hour cut-off.

	time in the first 2 days after childbirth.		
Iron supplementation (lactating women) (W)	No	Percentage of lactating women who received any iron-containing supplement within 6 months after childbirth.	• Denominator issues<br>○ Determining if women are lactating<br>• Define supplement type (including if IFA/MMN/iron only, tablet or syrup)<br>• Decide whether to include questions on quantity of pills/syrup received<br>• Consider additional indicators to capture consumption of iron received.
**CHILDHOOD PREVENTION AND PROMOTION**			
Support for exclusive and continued breastfeeding (L, W)	Yes, DHS 7 and MICS 6. Survey questions are included in the post-natal care module so time period is limited to any time in the first 2 days after childbirth. Support may be needed later on during infancy.	Percentage of women with a child 0-6 months of age who received advice/information from a health care provider or community worker within 1 month after childbirth; and/or Percentage of women with a child 0-6 months of age who were observed BF by a health provider or community. *Additional time periods after childbirth may be added depending on country/context-specific services.*	• Need to define time frame for when the observation occurred (e.g., within a month of birth)<br>• Should this only be asked for women with children 0-6 months old? (focus on exclusive BF as key indicator)<br>• For observation/ support requires that woman is still lactating but all women should receive information<br>• Assess whether lactation support should be asked about and if so, how
Counseling for exclusive and continued breastfeeding (L, W)	Yes, DHS 7 and MICS 6 Survey questions are included in the post-natal care module so time period is limited to any time in the first 2 days after childbirth. Information/counseling may be needed later on during infancy.	Percentage of women with a child 0-6 months of age who received information/counseling about exclusive breastfeeding from a health provider or community worker in the last 6 months.	• Define the time frame from childbirth (e.g., within 1 month, within the first 6 months, etc)<br>• Define the age range of the child (e.g, women with a child 0-6 months of age)<br>• Define list of health care providers
Counseling for complementary feeding (L, W)	No	Percentage of women with a child 6-24 months of age who received information from a provider about key components of complementary feeding within the previous X months:<br>• Timing of introduction of semi-solid & solid foods<br>• Diet diversity<br>• Other local messages?	• Define the recall period<br>• Need to adapt the key components to local protocols<br>• Define the list of providers
Food supplementation for complementary feeding in food insecure populations (L, W)	Yes, DHS 7. Questions ask only about ready-to-use supplemental foods with 7-day recall period. Questions are not currently located with the IYCF practice questions.	Percentage of children 6-23 months of age from food insecure populations who received any food supplements in the last X months	• Determine which children live in a food insecure population (define food insecurity at a population level)<br>• Decide if additional questions/indicators are needed that capture information about consumption of food supplements, and the frequency of consumption<br>○ Decide if a minimum duration of receipt/consumption should be set (i.e., consuming any complementary food may not be a meaningful indicator, but data on frequency may be informative)<br>• Define types of food/macronutrient supplement<br>• Age group and recall period need to align with local program recommendations
Iron supplementation in a population where children of 6-59 mo have 20% or higher prevalence of anemia (W)	No	Percentage of children (6-59 months) in a selected population who received any iron supplements in the X days before the survey.<br><br>Percentage of children (6-59 months) in a selected population who received at least X doses of iron supplements in the X days before the survey	• Denominator issue:<br>○ Identify populations of children ages 6-59 months that have 20% or higher prevalence of anemia<br>○ Consider national policy<br>• Decide if additional questions/indicators are needed that capture information about consumption of iron supplements<br>• Define the type of supplement<br>• Age group and the recall period need to align with local program recommendations

Vitamin A supplementation (high-dose)*** (L, W)	Yes, DHS 7.<br>Questions ask about a single dose in the previous 6 months.	Percentage of children (6-59 months) who received a high dose vitamin A supplement in the six months preceding the survey	• Timing of supplementation may not be exact (i.e., if two doses are given in a 12 month period, one may not have been given in the last 6 months)
Multiple micronutrient powders (MNP for anemia) in a population where children of this age have 20% or higher prevalence of anemia (L, Y)	Yes, DHS 7.<br>Questions use a 7-day recall period, which is limited to current consumption.	Percentage of children (6-59 m) in a selected population who received *any* MNP in the last X days prior to the survey<br><br>Percentage of children (6-59 m) in a selected population who received at least N doses of MNP in the last X days prior to the survey	*Same considerations as for iron supplementation in this age group*<br><br>• Confirm minimum definition of MNP (e.g. 3 or more with iron)
Preventive zinc supplementation (L)	No	Percentage of children (6-59 months) who received any preventive zinc supplementation in the X days before the survey	• Decide whether to include questions on quantity of pills/syrup received
**CHILDHOOD TREATMENT**			
Management of severe acute malnutrition (SAM) (L, W)	Yes, DHS 7.<br>Questions ask only about ready-to-use therapeutic foods with 7-day recall period. Challenge is on identifying the appropriate population of children (the denominator)	Percentage of children 6-59 months of age who are identified as having SAM that received treatment (a special food supplement)	• Correct measurement involves the following sequence of steps:<br>○ Child correctly screened for SAM<br>○ Child found to be suffering from SAM referred for follow-up<br>○ Child suffering from SAM receives special food supplement<br>• For facility assessments, documentation of the availability of screening equipment (e.g., MUAC) and treatment commodities, as well as a functioning referral system for in-patient and out-patient care for malnourished children.
Management of moderate acute malnutrition (MAM) (L, W)	Yes, DHS 7<br>Questions ask only about ready-to-use supplemental foods with 7-day recall period. Challenge is on identifying the appropriate population of children (the denominator)	Percentage of children 6-59 months of age who are identified as having MAM that received treatment (a special food supplement)	• Correct measurement involves the following sequence of steps:<br>○ Child correctly screened for MAM<br>○ Child found to be suffering from MAM referred for follow-up<br>○ Child suffering from MAM receives special food supplement.
Zinc supplementation with ORS for children with diarrhea (L, W)	Yes, DHS 7 and MICS 6.<br>Both survey programs use an aided recall of receipt of zinc.	Percentage of children who received zinc and ORS for an episode of diarrhea in the 2 weeks before the survey	• Age group needs to be determined, based on national policy

KEY: W = Recommended by WHO; L = Included in the Lancet Maternal and Child Nutrition Series, 2013

** “The preconception phase” includes interventions delivered during the time period prior to a first pregnancy and interpregnancy intervals as well as interventions provided to women of reproductive age and adolescent girls (ages 10-14) who do not eventually or ever become pregnant.

*** UNICEF includes in its databases the following indicator which is based upon a combination of survey and administrative data: Percentage of children ages 6-59 months who received two doses of vitamin A during the calendar year.
